# Overlapping Case of Advanced Systemic Sclerosis and IgG4-Related Disease after Autologous Hematopoietic Stem Cell Transplantation

**DOI:** 10.3390/medicina60030496

**Published:** 2024-03-18

**Authors:** Alisa Julija Dulko, Irena Butrimiene, Alma Cypiene, Valdas Peceliunas, Donatas Petroska, Ernesta Stankeviciene, Rita Rugiene

**Affiliations:** 1 Faculty of Medicine, Vilnius University, 03101 Vilnius, Lithuania; 2 Clinic of Rheumatology, Orthopaedics Traumatology and Reconstructive Surgery, Institute of Clinical Medicine, Faculty of Medicine, Vilnius University, 03101 Vilnius, Lithuania; 3 Department of Personalised Medicine, State Research Institute Centre for Innovative Medicine, 08406 Vilnius, Lithuania; 4 Hematology, Oncology and Transfusion Medicine Center, Vilnius University Hospital Santaros Klinikos, 08661 Vilnius, Lithuania; 5 Clinic of Internal Diseases, Family Medicine and Oncology, Institute of Clinical Medicine, Faculty of Medicine, Vilnius University, 03101 Vilnius, Lithuania; 6 Department of Pathology, Forensic Medicine and Pharmacology, Institute of Biomedical Sciences, Faculty of Medicine, Vilnius University, 03101 Vilnius, Lithuania; 7 Centre for Radiology and Nuclear Medicine, Vilnius University Hospital Santaros Klinikos, 08661 Vilnius, Lithuania

**Keywords:** IgG4-related disease, systemic sclerosis, overlap syndrome, autologous hematopoietic stem cell transplantation

## Abstract

Both scleroderma and immunoglobulin G4-related disease (IgG4-RD) are systemic fibro-inflammatory diseases characterised by lymphoplasmacytic infiltrates. IgG4-RD and systemic sclerosis (SSc) may share common pathophysiological mechanisms, but no examples of co-occurrence of the diseases have been found. Autologous haematopoietic stem cell transplantation (AHSCT) is implemented in selected rapidly progressive SSc with a high risk of organ failure. However, existing guidelines are based on clinical trials that do not represent the entire patient population and exclude critically ill patients with no therapeutic alternatives. Examples of AHSCT in IgG4-RD are absent. We report the case of a 44-year-old female patient with overlapping progressive diffuse SSc and sinonasal IgG4-RD. After 11 years of ineffective SSc treatment, AHSCT was performed. The 63-month follow-up showed a regression of SSc symptoms. AHSCT was not intended as treatment in the case of IgG4RD, although the first symptoms of the disease developed before transplantation. The sinus lesions progressed after AHSCT and remained indolent only after surgical treatment (bilateral ethmoidectomy, sphenoidotomy, intranasal buccal antrostomy), which allowed histopathological confirmation of IgG4-RD.

## 1. Introduction

Systemic sclerosis (SSc) is a heterogeneous, autoimmune connective tissue disease characterized by immune response changes, vasculopathy and progressive fibrosis [1]. Commonly, Raynaud’s phenomenon and skin fibrosis are early signs of the disease. As SSc progresses, internal organs such as the lungs, heart or kidneys can be affected. In 47.6% to 68% of cases, SSc-related complications, most commonly interstitial lung disease (ILD), lead to a lethal outcome [2,3]. Currently, cyclophosphamide (CYC) is the drug of choice for SSc-associated ILD. However, the data on CYC are contradictory. The treatment effect is often only partial, the disease progresses after discontinuation of the drug and long-term use is highly toxic [4,5,6]. In contrast, autologous hematopoietic stem cell transplantation (AHSCT) is more effective but associated with a higher risk [7,8,9]. 

IgG4-RD is a rare systemic fibro-inflammatory disease first recognized as a separate entity in 2003. It can affect any organ, causing a variety of symptoms [10,11]. Organ dysfunction develops through infiltration of tissue by lymphocytes and IgG4-secreting plasma cells, leading to inflammation, hypertrophy and progressive tissue fibrosis [10]. The aetiology of IgG4-RD disease is still unclear. Given the association with certain HLA variants, the overlap with other autoimmune diseases and the sensitivity to antirheumatic drugs, it is reasonable to consider IgG4-RD as a systemic autoimmune disease [12].

IgG4-RD of the head and neck with sinonasal involvement is very rare and is not included in the 2019 EULAR (European League Against Rheumatism) and ACR (American College of Rheumatology) classification criteria [13]. Sinonasal disease is increasingly recognized, with the first case presented in 2009 [14]. The clinical and radiological features of sinonasal IgG4-RD are non-specific. Pathological IgG4-RD masses in the sinuses can cause swelling and pain, and less commonly, visual disturbances. The maxillary sinuses are mostly affected with only local progression [14,15]. On CT, IgG4-RD appears as an irregular, heterogeneous mass with soft tissue density. Destruction of the sinus bone wall with infiltration of the surrounding structures and thickening of the nasal septum can also occur. In MRI, hypointense changes in the T2 sequence are disease specific [15]. The IgG4-RD must be distinguished from neoplastic, infectious and autoimmune diseases. A reliable correlation between clinical picture and histopathological findings is essential to confirm the diagnosis. Pathological laboratory findings are not specific and elevated plasma IgG4 levels are frequent but not always present and when found do not confirm the diagnosis [10]. The standard diagnostic test is histological examination of the affected tissue. Three main histological criteria are dense infiltration of IgG4-positive (IgG4+) plasma cells and lymphocytes, storiform fibrosis and obliterating phlebitis (at least two of which are required to confirm the diagnosis). The number of IgG4+ cells in immunohistological staining and the ratio of IgG4+ cells to total IgG cells >40% are of minor significance [13,16].

Overlap syndrome (OS) is an autoimmune disease in which the classification criteria of at least two connective tissue diseases are met. In total, 20–30% of SSc patients develop OS [17]. Cases of IgG4-RD and overlapping systemic autoimmune connective tissue diseases are known, but no examples of comorbidity of SSc and IgG4-RD have been reported [12,18].

## 2. Case Presentation

In January 2003, a 33-year-old female patient presented with one-year lasting Raynaud’s phenomenon, finger numbness and swelling. Her medical history included allergic rhinitis, obstructive bronchitis, bronchial asthma, septal and adenoid surgery. Elevated antinuclear antibody (ANA) titers and a skin biopsy led to the diagnosis of SSc. Discrete signs of fibrotic lung changes were seen on chest X-ray.

Initial treatment included aspirin and pentoxifylline followed by additional penicillamine and prednisolone. After an allergic reaction to penicillamine, azathioprine (AZA) was initiated. Despite three years of outpatient therapy, the disease manifested as CREST-Syndrome (calcinosis; Raynaud’s phenomenon; oesophageal dysfunction; sclerodactyly with ulceration; telangiectasia). The patient worked as a community nurse, cold working conditions during flood season aggravated the disease progression and led to disability.

In September 2006, the follow-up examination revealed whole body skin induration with reduced range of motion, microstomia, prominent labial wrinkles, and acro-osteolysis in the radiographs. The chest X-ray was normal. Despite daily therapy with AZA 150 mg, prednisolone 10 mg and regular alprostadil 20–40 μg daily infusions, the patient developed dyspnoea on exertion. In February 2007, pulmonary function tests revealed moderate airway obstruction. After CYC pulse therapy (12 infusions of 1 g), skin induration decreased slightly, poor digital perfusion as well as exertional dyspnoea persisted and osteolytic changes progressed. High-resolution computed tomography (HRCT) showed signs of pulmonary fibrosis. Methotrexate (MTX) 10–15 mg weekly administration was started in July 2009. In 2012, the patient developed supraclavicular tumorous calcinosis (5 cm Ø).

The condition continued to deteriorate, in January 2014, multidisciplinary team decided to perform AHSCT. At the time of hospitalisation, the patient’s overall condition was satisfactory (Table 1). The echocardiogram showed moderate left atrial dilatation, first-degree tricuspid regurgitation, sclerodegenerative mitral valve, delayed left ventricular relaxation, an ejection fraction of >55% and no pulmonary arterial hypertension (PAH). The MRI of the heart showed no focal lesions and no signs of pleural effusion.

During AHSCT, haematopoietic progenitor cells were mobilised with filgrastim. A total dose of 200 mg/kg CYC (50 mg/kg on days -5, -4, -3, -2) and antithymocytic globulin (0.5 mg/kg on day -5 and 1.5 mg/kg on days -4, -3, -2, -1) in combination with methylprednisolone were used for conditioning. A complication-free transplantation of cumulative 4.95 × 10^6^/kg CD34+ cells was performed. After stimulation with filgrastim, regeneration of granulopoiesis set in on day 11 after transplantation. The results of the treatment are presented in Table 1.

Before AHSCT, the patient was screened for chronic infectious disease and other contraindications for the transplantation. The patient reported hoarseness, bilateral tingling and numbness in the postauricular region; therefore, an otolaryngologist was consulted. CT scans revealed signs of chronic frontitis, ethmoiditis and sphenoiditis (thickened mucous membranes) and the right maxillary sinus was completely opacified with a soft tissue mass protruding into the subcutaneous fat through frontal wall bone destruction. The leftmaxillary sinus was almost completely opacified, with areas of increased mucosal density. Despite the pathological findings, due to the severity of the SSc and the unfavorable prognosis, AHSCT was performed and outpatient follow-up of sinonasal changes was recommended.

**Figure 1:**
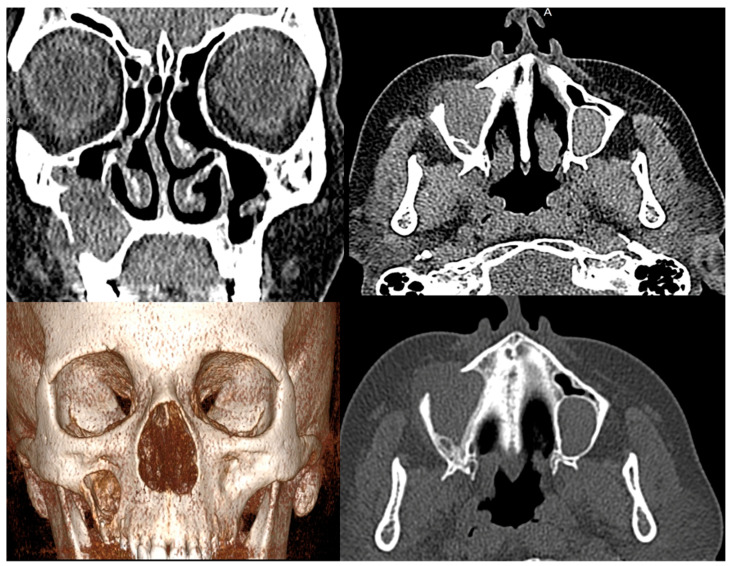
Preoperative (August 2018) computed tomography (CT) scans (soft tissue window, frontal and axial planes; volumetric reconstruction; bone window, axial plane) showing soft tissue masses filling almost the entire right maxillary sinus cavity, with bone destruction and protrusion into the soft tissues; a retention cyst in the left maxillary sinus.

In spring 2018, the patient developed discomfort and swelling in the right cheek. The CT scans were performed (Figure 1). In September 2018, MRI showed moderate progression of the lesions. Cystic, irregular, masses with slight peripheral accumulation of contrast and heterogeneous signal intensity (SI; lesion on the right: 2.9 × 2.3 × 2.3 cm, on T1 hypointense with hyperintense centre, on T2 hyperintense with hypointense centre; lesion on the left: 1.5 × 2.1 × 2.4 cm in T1 and T2 hyperintense, with higher SI insertion on T1, reduced SI on T2) were observed in both maxillary sinuses.

**Figure 2:**
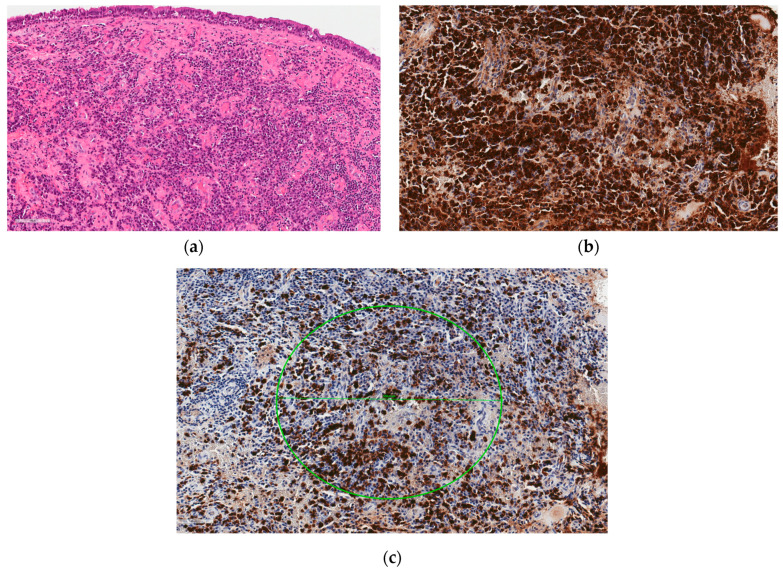
Hematoxylin and eosin staining (HE ×400) shows lymphoplasmacytic infiltration with dominating plasmocytes in the focally fibrosed (but not in storiform pattern) stroma of respiratory mucosa and increased amount of blood vessels with mild hialinization of walls (**a**); Immunostaining for IgG (IgG ×400) shows virtually all plasmocytes in mucosal stroma being positive (**b**); Immunostaining for IgG4 (IgG4 ×400) demonstraits abundance of IgG4-positive plasma cells (brown colour): encircled area contains >50 IgG4 positive plasmocytes and corresponds to a field of view of 0.24 mm^2^ (high power of field: objective ×40 and ocular 10×/22), the IgG4^+^/IgG^+^ plasma cell ratio is about 40% (**c**).

In January 2019, endoscopic bilateral ethmoidectomy, sphenoidotomy and intranasal buccal antrostomy were performed. The histopathological findings included spongiosa bone, fibrotic tissue, mucosal fragments covered with columnar epithelium, with a highly pronounced, abundant infiltration of plasmocytes, lymphocytes and polymorphonuclear cells, with dominant IgG-positive (IgG+) infiltration (Figure 2). The IgG4+/IgG+ plasma cell ratio was approximately 40%. It was only after the histopathological findings were analysed that IgG4-RD was suspected.

**Figure 3:**
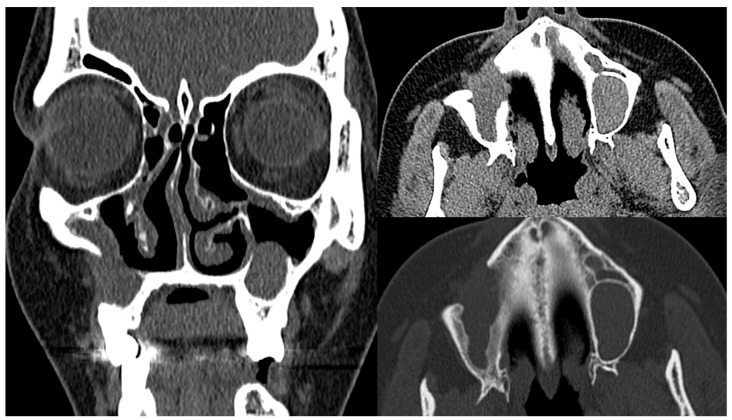
Post-operative (October 2019) CT scans (soft tissue window, frontal and axial planes; bone window, axial plane) showing reduced volume of the right maxillary sinus with post-operative changes, basal consolidation of the tissue and residual bone destruction. The lesions of the left maxillary sinus remained without dynamics.

At the post-operative follow-up examination in October 2019, the patient had no otolarygological complaints. The CT scans showed no post-operative progression of the sinonasal changes (Figure 3). In the IgG fraction analysis, the IgG4+ values were elevated, reaching 1.466 g/L (normal range 0.08–1.40 g/L). During the follow-up examinations in November 2021 and October 2023, the patient had no clinical symptoms related to the sinonasal changes and the CT scans showed no progression. The IgG4+ values were gradually increasing: 1.555 g/L in November 2021 and 1.664 g/L in October 2023. As there was no progression of the sinonasal changes and no relevant clinical symptoms after the surgical treatment, no further immunosuppressive treatment was initiated.

## 3. Discussion

The guidelines recommend AHSCT in selected patients with severe, rapidly progressive, refractory SSc in the first 4 to 5 years after diagnosis [19,20,21]. The approach is based on the results of a cohort study and three randomized controlled trials comparing different AHSCT regimens with CYC pulse therapy. The mean disease duration before transplantation in randomized controlled trials was 18.5 months, while the longest duration was 38 months. In the cohort study (NISSC1), the mean disease duration was longer (23.8 months), with the longest period before transplantation being 103.7 months [7,8,9,22]. The presented patient underwent effective AHSCT almost 12 years after the onset of the first SSc symptoms and 11 years (133 months) after diagnosis. This is an example of effective AHSCT in the late-stage SSc. However, some important advances have been made in recent years. New therapies (e.g., mycophenolate mofetil, nintedanib, rituximab (RTX) and tocilizumab for the treatment of the key disease manifestations) have become available and accepted by regulatory authorities. Hence, further research will be needed to investigate the role of AHSCT in the emerging context [23].

In the case presented, the presence of SSc led to a degree of uncertainty in the IgG4-RD diagnosis. The patient developed nonspecific but characteristic symptoms of sinonasal IgG4-RD. CT imaging was consistent with IgG4-RD, but nonspecific. MRI images also showed nonspecific cystic changes with heterogeneous signal intensity, without characteristic hypointense changes found in the T2 sequence [15]. Two findings necessary for confirmation of the histopathologic diagnosis were observed: fibrous tissue and a highly pronounced IgG+ and IgG4+ plasmocytic infiltration. Obliterative phlebitis was not described [16]. The absence of storiform pattern tissue fibrosis typical of IgG4 further complicated the clinical interpretation of the histopathological findings, since both IgG4-RD and scleroderma are characterized by lymphoplasmacytic infiltrates and fibrosis. In addition, IgG4+ plasma cell infiltrates were also detected in SSc skin samples, although the number of IgG4+ cells and the ratio of IgG+ to IgG4+ were insignificant [24]. Nevertheless, the role of IgG4 in the pathogenesis of IgG4-RD is still unclear, as this IgG subtype is anti-inflammatory and has little ability to activate the complement system [12]. Observation of the clinical course increased certainty that the clinical symptoms were expressions of two different nosological entities. After transplantation, the sinus lesions progressed, while the treatment was effective for the SSc-specific lesions. On the other hand, after surgical removal of the sinus lesions and without IgG4-RD-specific treatment, no progression of the disease was observed within 9 months, which may be due to the transplantation.

A plausible pathogenetic pathway for the manifestation of the overlap syndrome may be that both diseases are driven by an interplay of T and B cells, with subsets of T cells and CD4+ cytotoxic T lymphocytes (CTLs) playing a dominant role. T helper 2 cells in SSc and T follicular helper cells in IgG4-RD cells have overlapping phenotypic and functional characteristics. They express interleukin-4 and thus promote B-cell differentiation. Meanwhile, CTLs secreting interferon-γ, interleukin-1β, and tumor growth factor-β play important roles in the process of tissue damage and fibrosis [1,12,25]. T lymphocytes are stimulated by the gut microflora, which is remarkably similar in IgG4-RD and SSc patients and differs from healthy populations (e.g., more opportunistic pathogens and pro-inflammatory bacteria), suggesting a common pathological pathway [26]. Further studies on the gut microbiome are needed to confirm this hypothesis.

The fact that infiltrative sinonasal lesions developed during monotherapy with immunosuppressive drugs (MTX, AZA, CYC) and progressed after transplantation may suggest that the given treatment is ineffective in sinonasal IgG4-RD. However, without immunosuppressive treatment and AHSCT, a more aggressive course of the disease might have been possible. Further monitoring of the patient is required to confirm progression-free survival. Although sinonasal IgG4-RD is not malignant, systemic evaluation and timely treatment are necessary to prevent irreversible fibrous lesions. With steroids being the first-line therapy, RTX or AZA are suggested in case of an inadequate response or relapse [14]. In addition, RTX could be a therapy of choice for post-transplant systemic sclerosis [27]. In this respect, if required, RTX could have been proposed for further treatment of the patient.

## 4. Conclusions

This is an instance of systemic sclerosis and sinonasal IgG4-RD overlap syndrome with successful autologous hematopoietic stem cell transplantation in the late-stage progressive diffuse systemic sclerosis. The diagnostic of IgG4-RD is challenging in the case of overlapping systemic sclerosis. Although IgG4-RD does not belong to the spectrum of scleroderma diseases, a possible aetiopathogenetic microbiological link between the diseases has been identified. In this clinical example, IgG4-RD developed and progressed despite standard immunosuppressive therapy for systemic sclerosis and autologous hematopoietic stem cell transplantation, however, after surgical treatment and possibly in association with previous therapies, the clinical course of IgG4-RD remained indolent. Further monitoring of the patient is needed to confirm the validity of the conclusions.

## Figures and Tables

**Table 1:** Pre- and post-transplant assessment results.

	Before AHSCT	After AHSCT		
Date (Time after AHSCT)	2014 01	2014 09 (8 mo.)	2015 04 (15 mo.)	2019 04 (63 mo.)
Hand injury, Raynaud’s syndrome, skin conditions	Advanced sclerodactyly with flexural contractures, osteolysis, deep scarring, cutaneous fibrosis over the whole body, restricted movement	Softer hands and body skin, greater range of movement	Skin induration is reduced all over the body, acral contractures are reduced, wrinkles appear on the face, the amplitude of the yawning increases	Raynaud’s syndrome persistence, mild cutaneous induration; sclerodactyly with flexural contractures, multiple scars, osteolysis of the fingertips (overall positive dynamic)
DLCO, %,	67% mild reduction	61% mild reduction	63% mild reduction	73% mild reduction
Pulmonary function test assessment	FVC 2.6 (81% Ref 3.23), FEV_1_ 1.77 (64% Ref 2.78), FEV_1_/FVC 68% Ref 81%	FVC 2.71 (84% Ref 3.23), FEV_1_ 1.75 (63% Ref 2.78), FEV_1_/FVC 65% Ref 81%	FVC 2.6 (81% Ref 3.21), FEV_1_ 1,74 (64% Ref 2.75), FEV_1_/FVC 67% Ref 81%	FVC 2.39 (77% Ref 3.1), FEV_1_ 1.68 (63% Ref 2.65), FEV_1_/FVC 70% Ref 80%
The patient subjectively assessed her condition	Worsening	50–60% improvement	90% improvement	30% improvement
VAS	3	1	1	3
mRSS	40	34	30	23
HRCT scan (semi-quantitative analysis according to Warrick, 0–30 points)	14 (GGO, marginal pleural irregularities, intersegmental and subpleural lines)	13 (slightly less of GGO)	12 (GGO, pleural thickenings, subpleural lines and solitary subpleural cysts)	12
Capillaroscopy	Abundant avascular zones, isolated giant capillaries, signs of angiogenesis, marked fibrosis	Increased capillary density and isolated haemorrhages are observed as ischaemia and fibrosis decrease	In dynamics, increasing capillary density, decreasing areas of avascular zones, increasing number of giant capillaries	
	Anti-Scl-70 and anti-dsDNR positive			Anti-Scl-70 and anti-dsDNR negative

AHSCT—autologous haematopoietic stem cell transplantation; DLCO—diffusing capacity for carbon monoxide (normal range 77–100%); FVC—forced vital capacity; Ref—reference values; FEV1 forced expiratory volume in 1 s; VAS—visual analog scale for pain (0–10 cm, 0 cm corresponds to the lowest pain intensity); mRSS—modified Rodnan skin score (0–51, 0 corresponds to the condition with the fewest skin lesions); HRCT—high-resolution computed tomography; GGO- ground-glass opacification; anti-dsDNA—anti-double stranded DNA, anti-Scl-70—antibodies against topoisomerase I (norm negative).

## Data Availability

No new data were created or analyzed in this study. Data are contained within the article.
